# Characterization of Cauliflower OR Mutant Variants

**DOI:** 10.3389/fpls.2019.01716

**Published:** 2020-01-21

**Authors:** Ralf Welsch, Xiangjun Zhou, Julian Koschmieder, Tim Schlossarek, Hui Yuan, Tianhu Sun, Li Li

**Affiliations:** ^1^ Faculty of Biology II, University of Freiburg, Freiburg, Germany; ^2^ Robert W. Holley Center for Agriculture and Health, Agricultural Research Service, US Department of Agriculture, Cornell University, Ithaca, NY, United States; ^3^ Plant Breeding and Genetics Section, School of Integrative Plant Science, Cornell University, Ithaca, NY, United States

**Keywords:** protein variants, carotenoids, cauliflower, phytoene synthase (PSY), protein stability and folding, dimerization

## Abstract

Cauliflower *Orange* (*Or*) mutant is characterized by high level of β-carotene in its curd. *Or* mutation affects the OR protein that was shown to be involved in the posttranslational control of phytoene synthase (PSY), a major rate-limiting enzyme of carotenoid biosynthesis, and in maintaining PSY proteostasis with the plastid Clp protease system. A transposon integration into the cauliflower wild-type *Or* gene (*BoOR-wt*) results in the formation of three differently spliced transcripts. One of them is characterized by insertion (*BoOR-Ins*), while the other two have exon-skipping deletions (*BoOR-Del* and *BoOR-LD*). We investigated the properties of individual BoOR variants and examined their effects on carotenoid accumulation. Using the yeast split-ubiquitin system, we showed that all variants were able to form OR dimers except BoOR-LD. The deletion in BoOR-LD eliminated the first of two adjacent transmembrane domains and was predicted to result in a misplacement of the C-terminal zinc finger domain to the opposite side of membrane, thus preventing OR dimerization. As interaction with PSY is mediated by the N-terminus of BoOR, which remains unaffected after splicing, all BoOR variants including BoOR-LD maintained interactions with PSY. Expression of individual *BoOR* mutant variants in Arabidopsis revealed that their protein stability varied greatly. While expression of *BoOR-Del* and *BoOR-Ins* resulted in increased BoOR protein levels as BoOR-wt, minimal amounts of BoOR-LD protein accumulated. Carotenoid accumulation showed correlated changes in calli of Arabidopsis expressing these variants. Furthermore, we found that OR also functions in *E. coli* to increase the proportion of native, enzymatically active PSY from plants upon co-expression, but not of bacterial phytoene synthase CrtB. Taken together, these results suggest that OR dimerization is required for OR stability *in planta* and that the simultaneous presence of PSY interaction-domains in both OR and PSY proteins is required for the holdase function of OR. The more pronounced effect of simultaneous expression of all *BoOR* variants in cauliflower *Or* mutant compared with individual overexpression on carotenoid accumulation suggests an enhanced activity with possible formation of various BoOR heterodimers.

## Introduction

Carotenoids are a group of structurally diverse natural pigments, which have multifaceted functions in photosynthesis, phytohormone production, and generation of signaling molecules for plant growth and development (Cazzonelli and Pogson, 2010; Nisar et al., 2015; Yuan et al., 2015b; Rodriguez-Concepcion et al., 2018; Sun et al., 2018; Wurtzel, 2019). Being isoprenoid compounds, carotenoids are composed of the common building block isopentenyl diphosphate, like a large array of different isoprenoids, such as tocopherols, gibberellins, and chlorophylls. The initial specific reaction leading to the formation of different carotenoids in plastids is the condensation of two molecules of the C20 geranylgeranyl pyrophosphate to phytoene, catalyzed by the enzyme phytoene synthase (PSY). Subsequent desaturation reactions catalyzed by phytoene desaturase (PDS) and ζ-carotene desaturase (ZDS), as well as *cis-trans*-isomerization by carotenoid isomerase (CrtISO) and ζ-carotene isomerase (Z-ISO), result in the synthesis of the red-colored lycopene. Cyclization of lycopene by two different lycopene cyclases generates α- and β-carotene. Hydroxylations and epoxidations produce different xanthophylls in the carotenoid biosynthesis pathway.

Phytoene synthase is considered the major rate-limiting enzyme for the pathway and is therefore subjected to regulation at various levels (Sun and Li, 2020). Its transcript is induced by various environmental stimuli, such as light and abiotic stresses, mirroring increased carotenoid demands for the formation of the photosynthetic apparatus and/or abscisic acid biosynthesis (von Lintig et al., 1997; Welsch et al., 2008; Rodríguez-Villalón et al., 2009; Toledo-Ortiz et al., 2010). Moreover, posttranscriptional regulation of PSY translation in Arabidopsis occurs *via* its 5'UTR and dynamically adjusts PSY protein amounts to carotenoid levels in different tissues (Álvarez et al., 2016). PSY was also shown to interact with the upstream substrate-delivering enzyme GGPP synthase (GGPS) and acts as a constituent of metabolons containing up- and downstream enzymes to provide an additional possibility for regulation at the level of protein-protein interaction (Maudinas et al., 1977; Fraser et al., 2000; Ruiz-Sola et al., 2016; Camagna et al., 2019). Its evolutionary conserved features for activity across various organisms were recently analyzed (Cao et al., 2019). As PSY protein level is crucial for overall pathway activity and carotenoid amounts, a tight control of PSY proteostasis is expected. One of the major proteins regulating PSY protein level is the ORANGE (OR) protein. OR was found to regulate PSY protein stability *via* direct protein-protein interaction with PSY and by counterbalancing with the plastid-localized protein degradation machinery, the Clp protease complex, to maintain PSY proteostasis and fine-tune carotenogenesis (Zhou et al., 2015; Chayut et al., 2017; Welsch et al., 2018).

The *OR* gene was originally discovered as the one responsible for the orange-colored curd in a natural cauliflower mutant and later identified as a DnaJ-like cysteine-rich domain-containing protein (Li et al., 2001; Lu et al., 2006). Recently, it was found that the natural variation of the *OR* gene with a “golden SNP” defines melon fruit flesh color in a broad germplasm collection and governs β-carotene accumulation in melon fruit (Tzuri et al., 2015; Chayut et al., 2017). The “golden SNP” was demonstrated to alter the ability of *OR* for high levels of carotenoid accumulation (Yuan et al., 2015a; Kim et al., 2019). A recent study reveals that *OR* with a nonsynonymous mutation is also associated with carotenoid presence in carrot roots (Ellison et al., 2018). While ectopic expression of a wild-type *OR* gene increases carotenoid level (Bai et al., 2016; Park et al., 2016; Berman et al., 2017), likely due to its posttranslational upregulation of PSY protein level and activity, expression of either cauliflower *OR* mutant allele or an *OR* variant mimicking the “golden SNP” present in melon greatly promotes carotenoid accumulation in a number of plant species (Lopez et al., 2008; Yuan et al., 2015a; Kim et al., 2019; Yazdani et al., 2019). Interestingly, *OR* was recently found to regulate chloroplast biogenesis (Sun et al., 2019) and the expression of wild-type *OR* in sweetpotato and Arabidopsis lines enhances plant resistance to heat and oxidative stress treatments (Park et al., 2016; Kang et al., 2017; Kim et al., 2019).

In contrast to the melon *OR* with a single SNP that changes its capacity in inducing β-carotene accumulation (Tzuri et al., 2015), the mutation in cauliflower *Or* gene (*BoOR*-mut) is caused by a transposon integration within *BoOR.* This results in three different in-frame splicing variants to generate insertion and deletion *BoOR* variants (Lu et al., 2006). They encode one insertion carrying 13 additional amino acids from the transposon footprint (BoOR-Ins) and two deletions eliminating 13 and 42 amino acids but containing seven footprint amino acids (BoOR-Del and BoOR-LD), respectively (Lu et al., 2006). Remarkably, expression of the cauliflower *OR* mutant allele (*BoOR-mut*) carrying the transposon generates transgenic potatoes with high levels of carotenoid accumulation (Lopez et al., 2008; Li et al., 2012). However, when individual *BoOR* variants were expressed in cauliflower, none of these lines showed a phenotype similar to the cauliflower *Or* mutant (Lu et al., 2006).

The molecular mechanism underlying high carotenoid content in the cauliflower *Or* mutant as well as in the *BoOR-mut* overexpressing plants remains to be fully elucidated. To further examine the action of OR, we investigated the individual BoOR variants in detail. Our results suggest largely different properties provoked by different insertions and deletions in the OR variants, which are likely due to the dislocation of domains responsible for OR dimerization and PSY interaction.

## Materials and Methods

### Transmembrane Topology Prediction

Transmembrane domains and topology of different BoOR variants were predicted using the Phobius online tool (http://phobius.sbc.su.se/; Kall et al., 2007). Amino acid alignments were performed with Geneious (Biomatters).

### Yeast Two-Hybrid System

The split-ubiquitin system was used as previously described (Obrdlik et al., 2004; Welsch et al., 2018). Transit peptides of BoOR variants were predicted by ChloroP (Emanuelsson et al., 1999). cDNAs of *BoOR* variants without the transit peptide sequences were cloned into *pNXgate* in THY.AP4 to express fusion proteins with the N-terminal moiety of ubiquitin (Nub) and mated with cDNAs cloned in the vector *pmetY-cub* for fusion proteins with the C-terminal moiety of ubiquitin (Cub) present in THY.AP5 (for primers, see **Supplemental Table S1**). The yeast split-ubiquitin constructs for *BoOR-wt*, *AtOR* and *AtPSY* were used from previous work (Zhou et al., 2015). The resulting diploid cells were cultured in the synthetic complete medium lacking Leu and Trp. Interaction growth tests were performed on synthetic minimal agar, supplemented with 150 µM and 1 mM Met to reduce background activation of reporter genes. For β-galactosidase assays, yeast strains were grown overnight in synthetic complete medium supplemented with adenine and His at 28°C. β-Galactosidase activity was determined with ortho-nitrophenyl-β-galactoside (oNPG) as substrate in biological triplicates as previously described (Chayut et al., 2017) and expressed relative to cell density measured at OD_600nm_. Significance was determined by a Student's *t*-test.

### Protein Extraction and Western Blot Analysis

Proteins from yeast cells were extracted as previously described (Wang et al., 2004). Cells were lysed in 1.85 M NaOH (50 µl per 3 OD_600nm_ units), incubated on ice for 10 min, and an equal volume of 50% (w/v) trichloroacetic acid was added. Following centrifugation for 5 min, the pellet was suspended in 50 µl of SDS sample buffer containing 8 M urea and 20 µl 1 M Tris, and incubated for 1.5 h at 37°C before loading to gel. *E. coli* subfractions were prepared as published (Hundle et al., 1992). Bacterial cells from 50 ml culture were lysed in 10 ml phosphate-buffered saline (140 mM NaCl, 2.7 mM KCl, 10 mM Na_2_HPO_4_, 1.8 mM NaH_2_PO_4_, pH 7.4) by two passages through the French Press. Non-broken cells were pelleted by centrifugation at 3,100 g for 5 min and discarded. Inclusion bodies were pelleted from 7 ml of the supernatant (“lysate”) by centrifugation for 15 min at 13,000 g and membranes after re-centrifugation of the supernatant for 90 min at 100,000 g. Pellets were resuspended in 200 µl SDS sample buffer and 20 µl each used for western blot analysis. Lysate with 60 µg of total protein was used. For protein extraction from Arabidopsis, approximately 0.5 g of leaf tissue was ground to fine power in liquid nitrogen, and 500 μl of the extraction buffer (50 mM HEPES/KOH, pH 7.5, 0.15 M NaCl, 0.5% [v/v] Triton X-100, 0.1% [v/v] Tween 20, and 1% protease inhibitor cocktail) was added to extract proteins. After centrifuged at 12,000 rpm for 10 min, the supernatants were transferred into new microcentrifuge tubes and used for western blot analysis.

Western blot analysis was performed as previously described (Welsch et al., 2018). The total protein concentrations in protein extracts were determined using the Bradford method. Proteins were separated by SDS-PAGE and blotted onto nitrocellulose membranes (Schleicher & Schuell, Keene, NH, USA). The efficiency of blotting or loading was examined by Ponceau S staining. Immunodetection was performed using anti-GFP (Santa Cruz Biotechnology, Dallas, Texas), anti-HA (Sigma), anti-BoOR (Lu et al., 2006), anti-AtPSY (Maass et al., 2009), and anti-CrtI (Schaub et al., 2005) antibodies. Polyclonal anti-CrtB antibodies were raised against recombinant 6xHis-CrtB. Signals were developed with Amersham ECL detection reagent (Thomas Scientific, Swedesboro, NJ) and captured on X-ray films.

### Generation of Transgenic Arabidopsis

In order to express BoOR-GFP fusion proteins, the cDNAs of *BoOR-wt*, *BoOR-Del*, *BoOR-Ins*, and *BoOR-LD* without stop codons were amplified by PCR (for primers, see **Supplemental Table S1**) and cloned upstream of *GFP* between *Eco*RI and *Nco*I sites in *pAVA393* (von Arnim et al., 1998). The chimeric genes were then subcloned into *pCAMBIA1300S* between *Kpn*I and *Sma*I sites (Zhou et al., 2010). After verification by sequencing, the plasmids were individually transferred into *Agrobacterium* GV3101 by electroporation, and transformed into Arabidopsis using floral dip transformation (Clough and Bent, 1998). The positive transformants were screened and used to generate homozygous transgenic lines.

### GFP Detection

GFP fluorescence signals in leaf surface cells of two-week-old transgenic Arabidopsis were analyzed by using Leica TCS SP5 laser scanning confocal microscope (Leica Microsystems, Exton, PA, USA) with excitation wavelength at 488 nm and emission wavelength at 500–520 nm.

### Induction of Seed-Derived Calli

Seed-derived calli from various *BoOR* expressing lines were generated as previously described (Schaub et al., 2018).

### RNA Extraction and qRT-PCR

Total RNA was extracted from Arabidopsis using Trizol reagent (Invitrogen, Carlsbad, CA). After treatment with RQ1 DNase (Promega, Madison, WI, USA) for 30 min to remove genomic DNA, RNA samples were converted into cDNA using iScript™ Reverse Transcription Supermix (Bio-Rad, Hercules, CA, USA). Quantitative real-time PCR (qRT-PCR) was conducted in an ABI 7500 real-time PCR system by using iTaq Universal SYBR Green Supermix (Bio-Rad). The thermal cycle involved 95°C for 3 min, and 40 cycles of 95°C 15 s and 60°C for 60 s, followed by melt curve analysis to verify the specificity of amplification. The relative gene expression was calculated by the ΔΔCt method with the Arabidopsis actin gene as an internal control (Lyi et al., 2007). Results were from two independent transgenic lines per construct with three technical replicates each.

### Heterologous Expression in *E. coli*

*E. coli* co-expressing Arabidopsis GGPS11, Arabidopsis PSY and *Pantoea ananatis* CrtI were generated as previously described (Camagna et al., 2019) using the vectors *pRSF-PSY*, *pACYC-GGPS11*, and *pCDF-CrtI*. cDNAs of *CrtB*, *AtOR*, *BoOR-wt*, and *BoOR* variants were subcloned in the vector *pETDuet*™*-1* (Novagen) using corresponding *pNXgate* and *pmetY-cub* vectors described above and the Gibson isothermal assembly method (Gibson et al., 2009). Similarly, N- and C-terminal moieties of AtOR, corresponding to amino acids 55 to 127 and 128 to 307 (accession number NM_203246), respectively, were subcloned in *pETDuet*™*-1*. Transformed *E. coli* cultures were induced for protein expression with 100 µM isopropyl β-D-1-thiogalactopyranoside (IPTG) and harvested during exponential growth phase at OD_600nm_ = 0.5.

### Carotenoid Analysis

Carotenoid extraction and analysis from callus samples were performed as previously described (Schaub et al., 2018). For the analysis, 10 milligrams of surface-sterilized seeds from each transgenic line were plated onto petri dishes (145 mm diameter) containing callus induction medium (4.33 g L^-1^ MS basal salts/KOH, pH 5.8, 3% [w/v] sucrose, 0.1% [v/v] Gamborg B5 vitamins, 0.5 mg L^-1^ 2,4-D, 2 mg L^-1^ indole-3-acetic acid, 0.5 mg L^-1^ 2-isopentenyladenine, 0.4% [w/v] phytagel), germinated under long-day conditions (16 h light/8 h dark, 26˚C) for 5 days, and incubated for 14 days in darkness. Calli sampled from three separate plates were considered as three biological replicates. For lycopene quantification from *E. coli* cells, bacterial pellets were resuspended in 300 µl acetone and centrifuged at 4,000 g for 5 min. The supernatant was transferred into a new tube and the pellet was re-extracted two times with 300 µl of acetone each. The combined acetone supernatants were evaporated to dryness. Lycopene was resuspended in petrol ether and quantified photometrically using ϵ_474nm_ = 185 230 L mol^−1^ cm^−1^. All experiments were performed with three biological replicates and significance was determined by a Student's *t*-test.

## Results

### BoOR Protein Structure Is Affected by Transposon Integration in the *BoOR-mut* Allele

**Figure 1:**
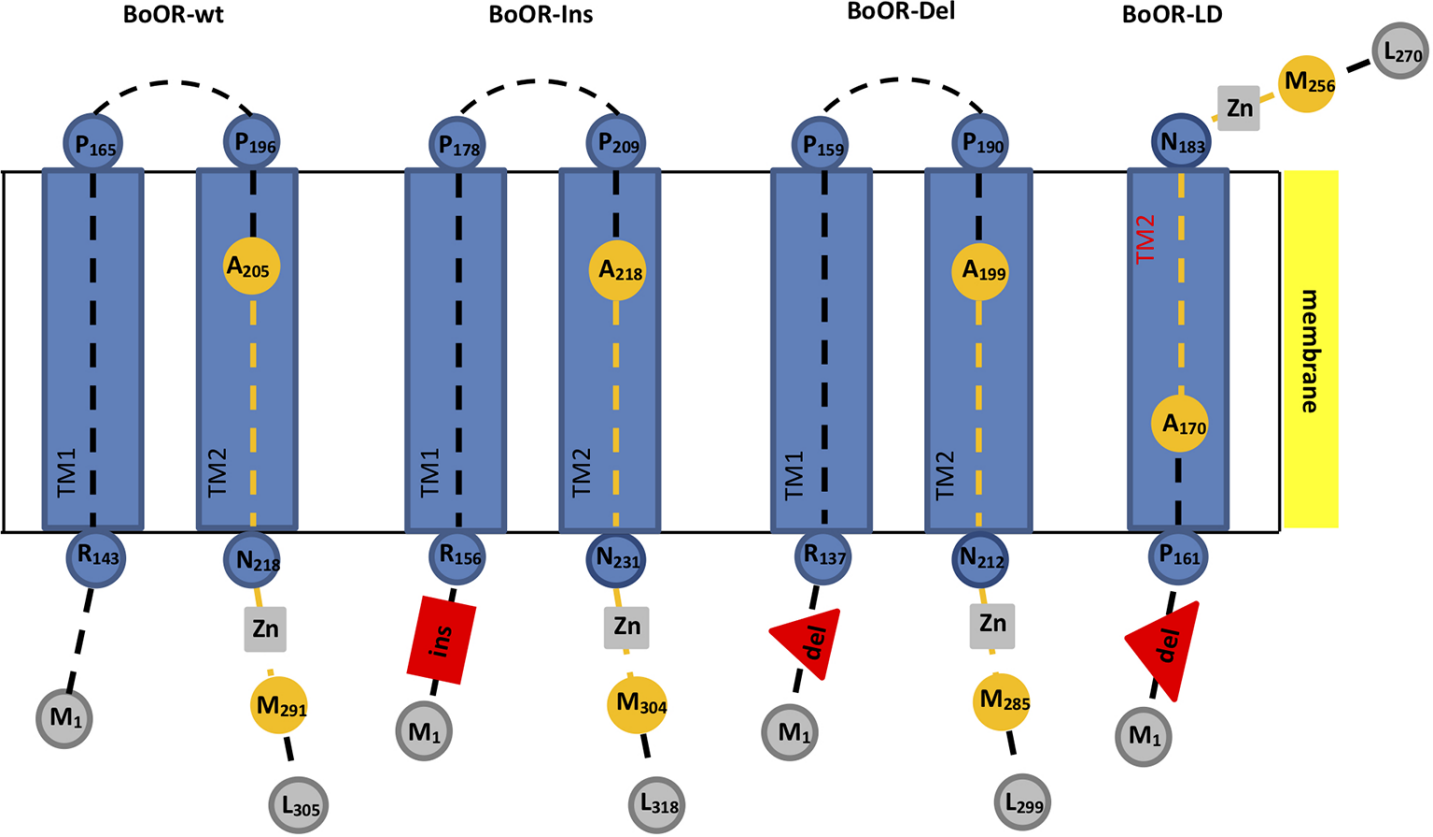
Transmembrane domain and topology prediction of different BoOR variants. Transmembrane domains and topology of different BoOR variants were predicted using the Phobius tool. Transmembrane domains (TM) are given as blue boxes. Peptide backbones are as dashed lines. Residues and their positions marking the beginning or end of a domain or the peptide backbone are given as circles. The DnaJ-like zinc finger domain which is involved in OR dimerization is highlighted in orange. It contains four CxxCxGxG repeats (grey boxes) with each two coordinating one zinc ion. Insertion and deletions are given in red. BoOR-Ins carries a 13 amino acid insertion (red box) while in BoOR-Del 13 amino acids are deleted (red triangle). In the large deletion variant BoOR-LD, a 42 amino acid stretch is deleted (large red triangle). In BoOR-Ins and BoOR-Del, topology, transmembrane domains and the DnaJ-like domain most likely remain unaffected. The large deletion in BoOR-LD fully removes TM1 while TM2 is likely assembled correctly (see red highlight). Accordingly, BoOR-LD protein topology is altered such that the C-terminus is positioned on the other side of the membrane and protein-protein interaction is hindered.

The OR protein shows highest homology to DnaJ-like molecular chaperones (Lu et al., 2006; Pulido and Leister, 2018). We performed a structural analysis of the cauliflower wild-type OR protein (BoOR-wt) using the Phobius tool (http://phobius.sbc.su.se/; Kall et al., 2007). The analysis revealed two putative transmembrane domains located in the central region of the protein sequence with a DnaJ-like Cys-rich zinc finger domain directly adjacent to the second transmembrane domain at the C-terminal half of the protein (**Figure 1**). The Cys-rich zinc finger domain carries four repeats of the motif CxxCxGxG, with two repeats separated only by three amino acids and spaced by 25 amino acids between both two tandem repeats (Lu et al., 2006). Yeast split-ubiquitin analysis with the N-terminal and the C-terminal OR moiety, the latter carrying the transmembrane and the zinc finger domains, indicates that OR dimerization is mediated solely by the C-terminal domain and does not require the N-terminal region (Zhou et al., 2015). In contrast, the N-terminal moiety is required for interaction with PSY to posttranslationally regulate PSY stability (Zhou et al., 2015; Chayut et al., 2017).

The cauliflower *Or* mutant is a gain-of-function mutation, characterized by a transposon insertion within *BoOR* creating the in-frame deletion and insertion variants BoOR-Ins, BoOR-Del, and BoOR-LD (Lu et al., 2006). In order to analyze whether these mutations affect the OR protein structure, we first performed a transmembrane prediction analysis for each variant (**Figure 1**, **Supplemental Figure S1**). The BoOR-Ins carries the 13 amino acid insertion E_123_LKSQNPNLLIQH_135_, which is located at the N-terminal side to the transmembrane domains and accordingly did not affect the prediction analysis of these domains. In BoOR-Del, the 13 amino acid sequence P_125_NFPSFIPFLPP_136_ in the wild-type protein is replaced by K_125_SQNPN_130_ at the N-terminal side to the transmembrane domains without affecting the predicted transmembrane domains. BoOR-LD contains a large deletion of the 42 amino acid sequence P_125_NFPSFIPFLPPLTAANLRVYYATCFSLIAGIILFGGLLAPT_166_ in the wild-type protein which is replaced by K_125_SQNPNL_131._ This large deletion fully removes the entire first transmembrane domain and was additionally predicted to slightly lower the likelihood for correct assembly of the second transmembrane domain, even though the primary sequence of this domain is unaltered. Accordingly, if the second transmembrane domain assembles correctly, the topology of BoOR-LD is altered such that the DnaJ-like zinc finger domain is on the other side of the membrane than it is in wild-type BoOR (see **Supplemental Figure S2** for amino acid alignment of BoOR variants).

### Deletion of One Transmembrane Domain in BoOR-LD Negatively Affects Dimerization Capacity

**Figure 2:**
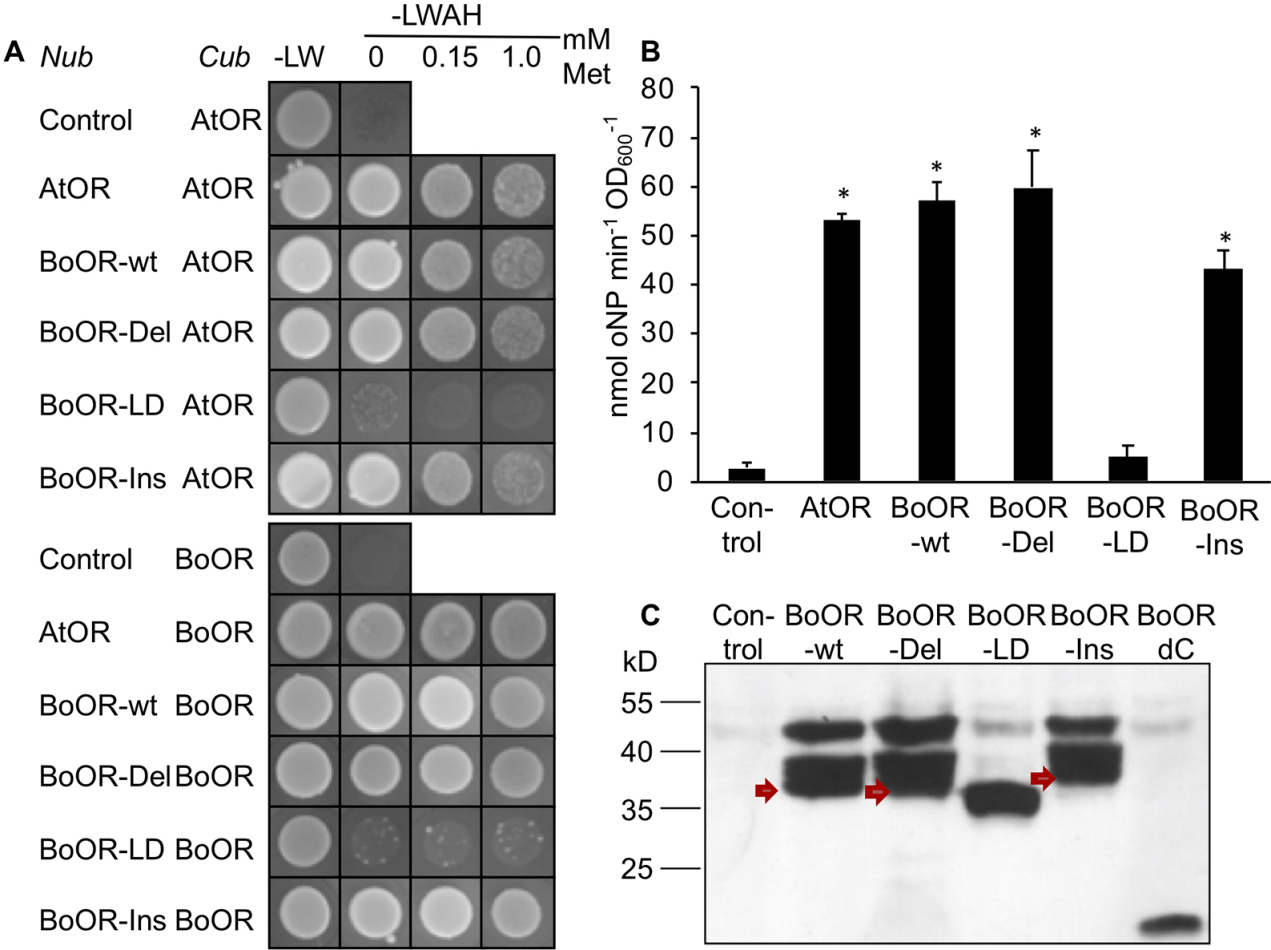
Analysis of interactions between various variants of OR. **(A)** Yeast strains expressing a C-terminal fusion of the C-terminus of ubiquitin (Cub) with Arabidopsis (AtOR) or cauliflower OR (BoOR) were combined with yeast expressing the N-terminus of ubiquitin (Nub) only (control) or N-terminal Nub fusions with AtOR, BoOR-wt or the BoOR mutant variants. Yeast combinations were spotted onto either nonselective (−LW) or fully selective medium plates (−LWAH) supplemented with methionine in the concentrations indicated. This represses expression of Cub fusion partner and indicates interaction strength. **(B)** β-Gal activity determined by oNPG assay of yeast strains coexpressing combinations with AtOR-Cub; the Nub-fused proteins of the combinations are indicated on the X-axis. Results are means ± SD from three biological replicates. *Significant difference to control combination (P < 0.05). **(C)** Nub-BoOR variant protein levels in AtOR-Cub combinations. Nub-BoOR variant proteins carried an N-terminal 3-HA tag and were detected using an anti-3-HA antibody. Note that only BoOR-LD migrates at its expected molecular mass of 34.5 kD with single band, while BoOR-wt (38.2 kD) as well as other variants show aberrant migrations with several bands at higher molecular masses than predicted sizes (BoOR-Ins: 39.7 kD; BoOR-del: 37.5 kD). Similarly, a C-terminal truncation of BoOR eliminating its transmembrane and zinc finger domains runs at the expected molecular mass of 19.2 kD. Nub indicates coexpression of the empty vector as negative control.

In order to investigate whether these structural changes affect OR dimerization (Zhou et al., 2015), we performed yeast split-ubiquitin assay with individual BoOR variants in combination with either the BoOR-wt or the homologous Arabidopsis OR protein (AtOR). In this system, one protein is fused to the C-terminal ubiquitin moiety (Cub) and an artificial transcription factor while the second protein is fused to the N-terminal ubiquitin moiety (Nub). Interaction of the two proteins in yeast membranes reconstitutes full-length ubiquitin, which is cleaved by cytosolic deubiquitinating enzymes releasing the transcription factor and subsequently induces expression of reporter genes (Snider et al., 2010). As shown in **Figure 2A**, both BoOR-wt and AtOR interacted with themselves and with each other to form homo- and heterodimers, respectively. Heterodimerization remained unaffected in the combinations with BoOR-Ins or BoOR-Del. However, no heterodimerization was observed with BoOR-LD when combined with either BoOR-wt or AtOR (**Figure 2A**). Quantification of interaction strength between BoOR variants and AtOR by oNPG assay confirmed these observations (**Figure 2B**). The assay revealed unchanged interaction strength for the combination of AtOR with BoOR-Del and only slightly reduced interaction with BoOR-Ins compared with the control combinations with wild-type OR proteins. However, the combination with BoOR-LD showed only the weak background activation of *lacZ* reporter gene as observed in combination with the empty vector control (**Figure 2B**).

In order to confirm that all BoOR variants were expressed in yeast co-expressing BoOR-wt, we performed western blot analysis using antibodies against the haemagglutinin (HA)-tag, which is present in the Nub moiety of all BoOR variants. As shown in **Figure 2C**, all variants were expressed with similar protein levels, but exhibited different migration behaviors in SDS-PAGE. In addition to one major band corresponding to the predicted molecular mass (BoOR-wt 38.2 kD, BoOR-Del 37.5 kD, and BoOR-Ins 39.7 kD), BoOR-wt and both variants, in which transmembrane/zinc-finger domains remained intact (BoOR-Ins and BoOR-Del), showed two aberrant bands corresponding to higher molecular masses (**Figure 2C**). Interestingly, only the BoOR-LD variant showed only one band corresponding to its predicted molecular mass (34.5 kD) (**Figure 2C**). These observations suggest a possible association of the two transmembrane domains with the aberrant migration behavior of BoOR-wt, BoOR-Del, and BoOR-Ins, which was lost with the elimination of one transmembrane domain in BoOR-LD. This was confirmed when the N-terminal BoOR moiety devoid of C-terminal domains (BoOR-dC) migrated with only its predicted molecular mass of 19.2 kD as HA-Nub fusion protein in yeast (**Figure 2C**).

### Deletion of One Transmembrane Domain in BoOR-LD Positively Affects Interaction With PSY

**Figure 3:**
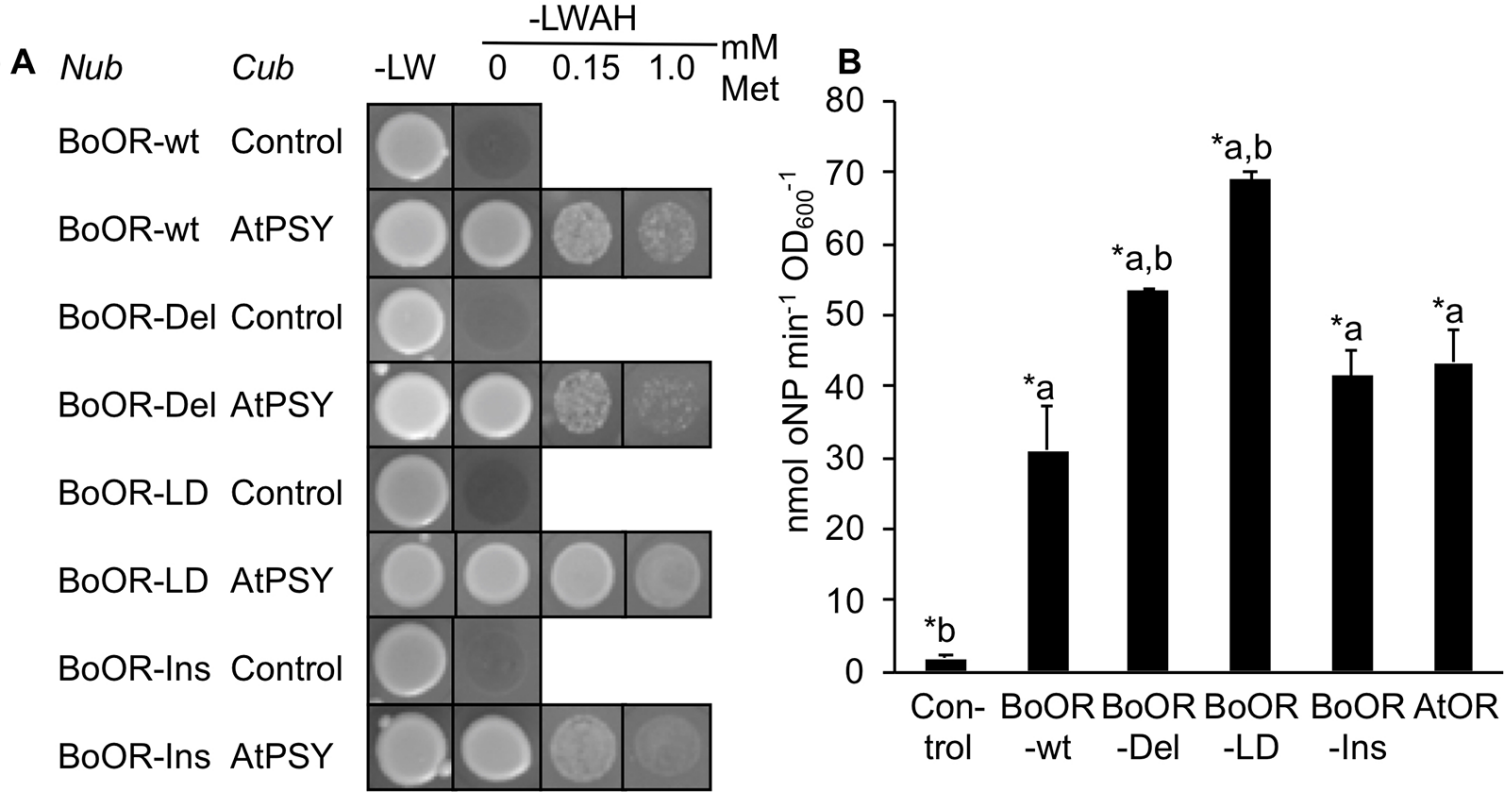
Interaction of BoOR mutant variants with Arabidopsis PSY. **(A)** Analysis of interactions between Arabidopsis PSY (AtPSY) and variants of cauliflower OR (BoOR-wt, BoOR-Del, BoOR-LD, BoOR-Ins). Yeast strains expressing a C-terminal Cub fusion with AtPSY or Cub only (control) were mated with yeast expressing N-terminal Nub fusions with BoOR-wt or the BoOR mutant variants. Yeast combinations were spotted onto either nonselective (−LW) or fully selective medium plates (−LWAH) supplemented with methionine in the concentrations indicated. This represses expression of Cub fusion partner, thus the continued growth in presence of methionine indicates high interaction strength. **(B)** β-Gal activity determined by oNPG assay of yeast strains coexpressing OR variants with AtPSY. Results are means ± SD from three biological replicates. *Significant difference (P < 0.05) with a, relative to Control and b, relative to BoOR-wt.

The interaction between OR and PSY occurs exclusively *via* the N-terminal moiety of OR (Zhou et al., 2015). To check whether interaction with PSY was affected in the BoOR variants, we performed additional split-ubiquitin assays using BoOR variants N-terminally fused with Nub and AtPSY C-terminally fused with Cub. Interestingly, while BoOR-Ins and BoOR-Del interaction strength with AtPSY was similar to that of the BoOR-wt control combination, BoOR-LD exhibited strongly increased interaction with AtPSY (**Figure 3A**). This was concluded from the observation that addition of increasing concentrations of methionine in selective growth plates which repressed expression of Cub fusion proteins retained growth of yeast co-expressing BoOR-LD and AtPSY (**Figure 3A**). Moreover, quantification of *lacZ* reporter gene expression by oNPG assay confirmed strong interaction strength in yeast co-expressing BoOR-LD and AtPSY (**Figure 3B**).

### Ectopic Expression of BoOR Variants in Arabidopsis

**Figure 4:**
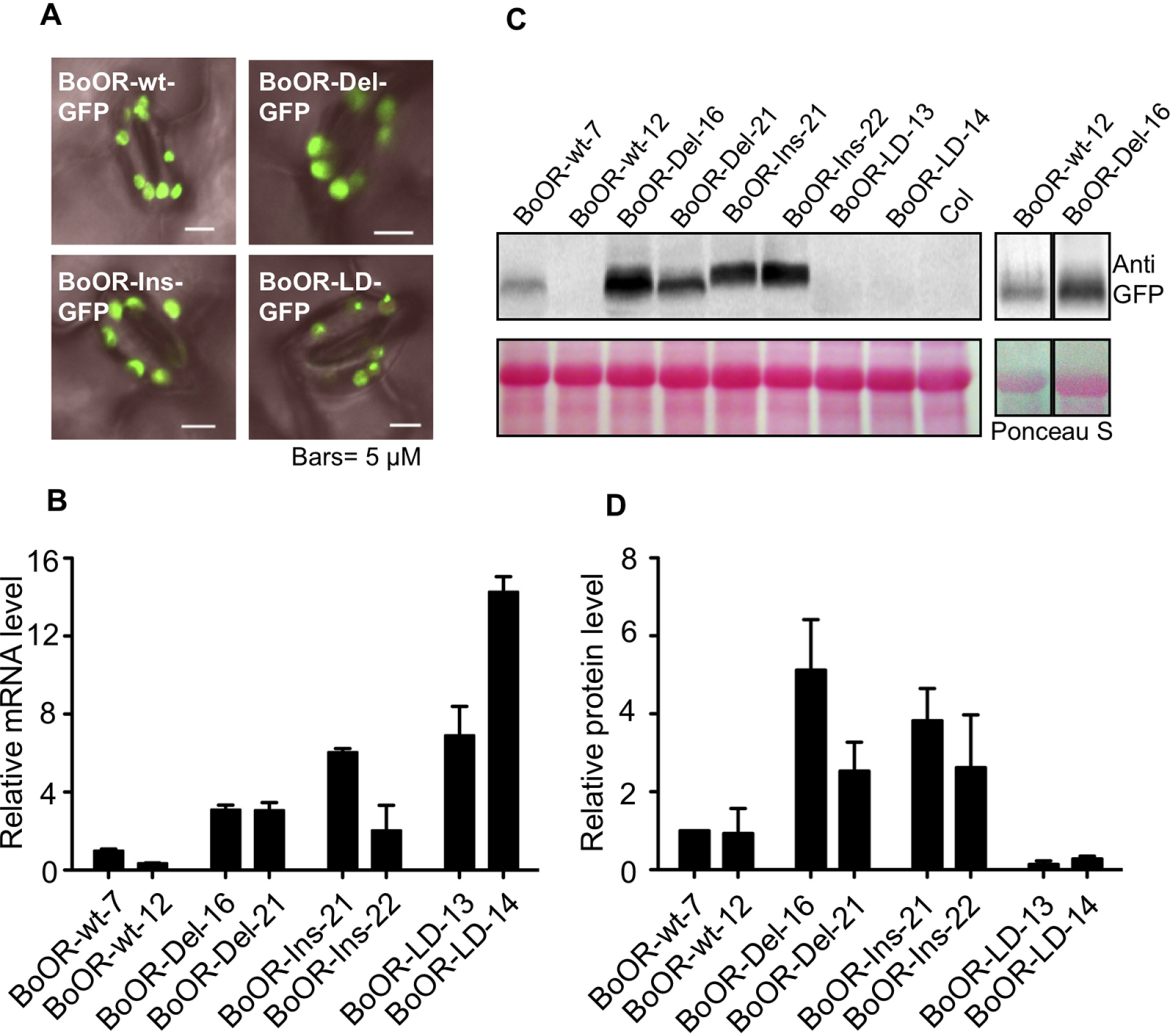
Overexpression of cauliflower OR variants in Arabidopsis. **(A)** Subcellular localizations of BoOR-wt and BoOR mutant variants were investigated by fluorescence microscopy in leaves. All variants were detected in chloroplasts, similarly to the wild-type BoOR-wt-GFP. **(B)** qRT-PCR analysis of *BoOR* expression in transgenic Arabidopsis lines expressing individual *BoOR* variants. Two independent homozygous transgenic lines per construct were examined. *BoOR* variant mRNA levels were normalized to actin and expressed relative to the levels detected in the line expressing *BoOR-wt-7*. **(C)** BoOR variant protein levels in leaves of transgenic Arabidopsis lines. Translational fusions of BoOR variants with GFP were detected using an anti-GFP antibody. Ponceau S staining of proteins after blotting is shown as loading control; the prominent band corresponds to RuBisCo large subunit (RBCL). **(D)** The OR protein levels from three western blots were quantified using ImageJ and normalized based on the amounts of protein loading. The relative OR protein levels were expressed as relative to that determined in BoOR-wt-7, which was set to 1.

As outlined above, BoOR variants are very likely to have different protein structures and membrane topology (**Figure 1**). To see whether the insertion and deletions in BoOR also affected their subcellular localization, we transformed various *BoOR-GFP* constructs into Arabidopsis and monitored GFP fluorescence in epidermal cells of young leaves. All three BoOR mutant variants as well as BoOR-wt were properly localized in chloroplasts (**Figure 4A**). The results indicate that the structure variations did not affect their plastid localization.

The BoOR variants exhibited variations regarding heterodimerization with wild-type OR and interaction with AtPSY (**Figures 2** and **3**). To investigate the potential impacts of these variations in plants, we examined transgenic Arabidopsis lines expressing individual *BoOR* mutant variants. The relative *OR* expression of two independent homozygous transgenic lines expressing each construct of *BoOR-wt* and individual *BoOR* mutant variants was examined (**Figure 4B**). We then investigated BoOR mutant variant protein levels in leaves of 4-week-old plants by western blot analysis (**Figures 4C, D**). As was the case in yeast (**Figure 2C**), the *BoOR* variant transgenic lines produced various sizes of proteins (**Figure 4C**). Noticeably, the protein level increases in the two independent transgenic lines of *BoOR-Del* or *BoOR-Ins* appeared proportional changes with their transcript levels compared with *BoOR-wt* lines (**Figures 2B** and **4D**). Interestingly, an opposed effect was observed for BoOR-LD. While transcript abundance was increased over 7-fold in the two independent *BoOR-LD* transgenic lines compared with the *BoOR-wt* expressing lines (**Figure 4B**), the BoOR-LD accumulated only in small amounts, suggesting increased protein instability in the *BoOR-LD* transgenic plants (**Figure 4D**).

### Effect of BoOR Variants on Carotenoid Accumulation in Arabidopsis

**Figure 5:**
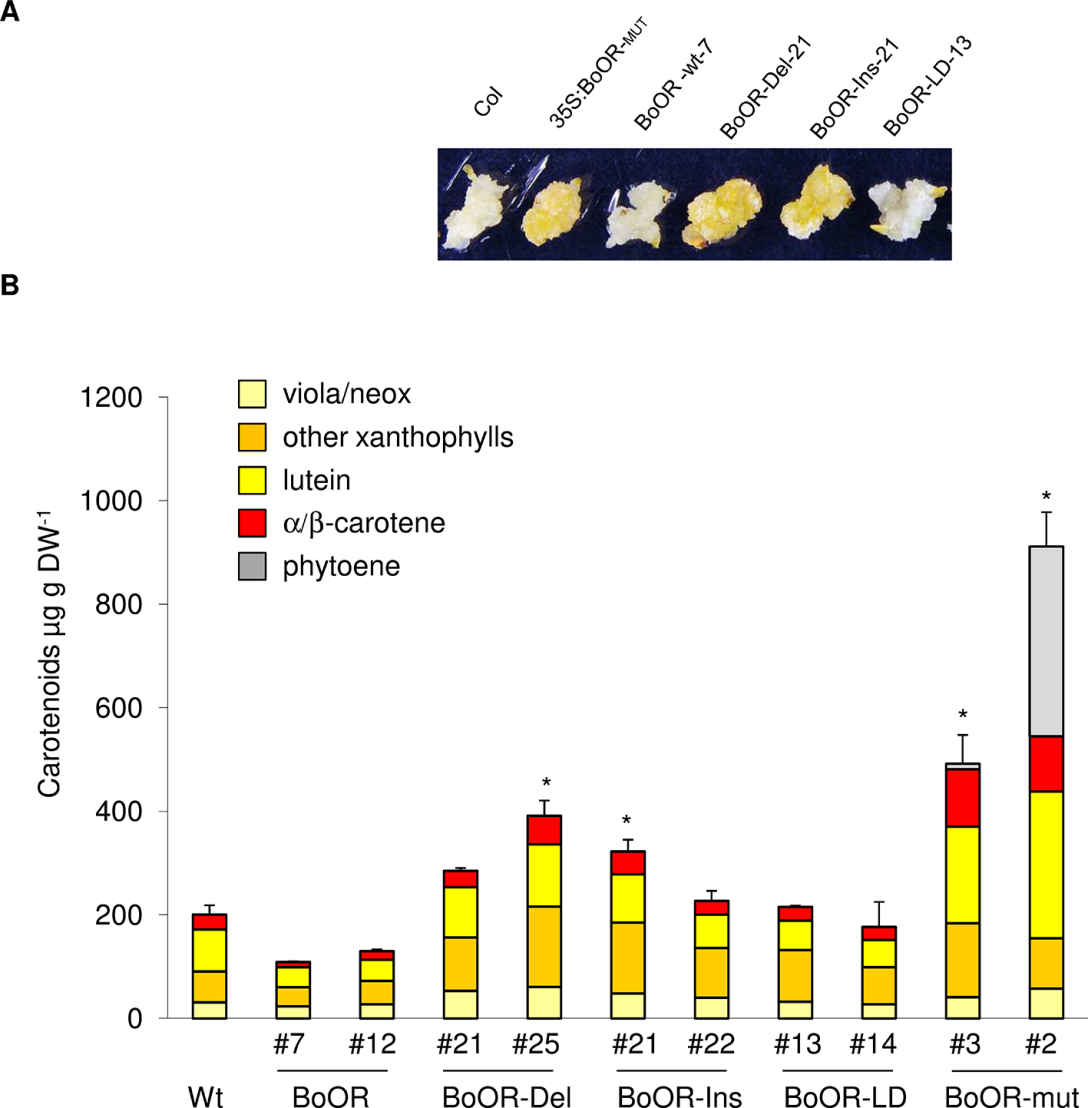
Carotenoid levels in Arabidopsis seed-derived calli expressing cauliflower OR variants. **(A)** Representative images from seed-derived calli of BoOR mutant variant expressing lines. **(B)** Carotenoid composition and content in BoOR variants in comparison with non-transgenic control. Total carotenoid amounts were expressed relative to wild-type sample. Calli generated from 10 mg of seeds in one petri dish plate were collected and considered as one biological replicate. Results are means ± SD from three biological replicates, *significant difference (P < 0.05).

The carotenoid pathway flux in green, photosynthetically active tissues and in non-green tissues is known to differ strongly (Maass et al., 2009; Lätari et al., 2015; Schaub et al., 2018). While green tissues often compensate for increased carotenoid pathway flux so that carotenoid amounts remain fairly constant, non-green tissues accumulate carotenoids upon increased pathway activity. Therefore, to investigate the effects of *BoOR* mutant variants on carotenoid amounts, we focused on non-green tissues. Seed-derived calli have been successfully established as suitable systems to study alterations in carotenoid pathway flux in Arabidopsis (Yuan et al., 2015a; Álvarez et al., 2016; Schaub et al., 2018). As shown in **Figure 5A**, calli expressing various *BoOR* variants exhibited different colors. Consistent with the callus phenotypes, up to 2-fold increases of total carotenoids were observed in the calli of the *BoOR-Del* and *BoOR-Ins* lines compared to the non-transgenic control (**Figure 5B**). The two *BoOR-LD* lines had similar carotenoid levels as the control. Calli from plants expressing *BoOR-wt* were pale and accumulated slightly less carotenoids than the non-transgenic control, which was however statistically insignificant (**Figure 5B**). Remarkably, the Arabidopsis line expressing the transposon-containing *BoOR* gene accumulated highest levels of carotenoids up to 600 µg DW^-1^ (**Figure 5B**). In these lines, the pathway intermediate phytoene accumulated, which indicates high pathway flux increases (Maass et al., 2009).

### Analysis of BoOR-PSY Interaction in a Heterologous Bacterial System

**Figure 6:**
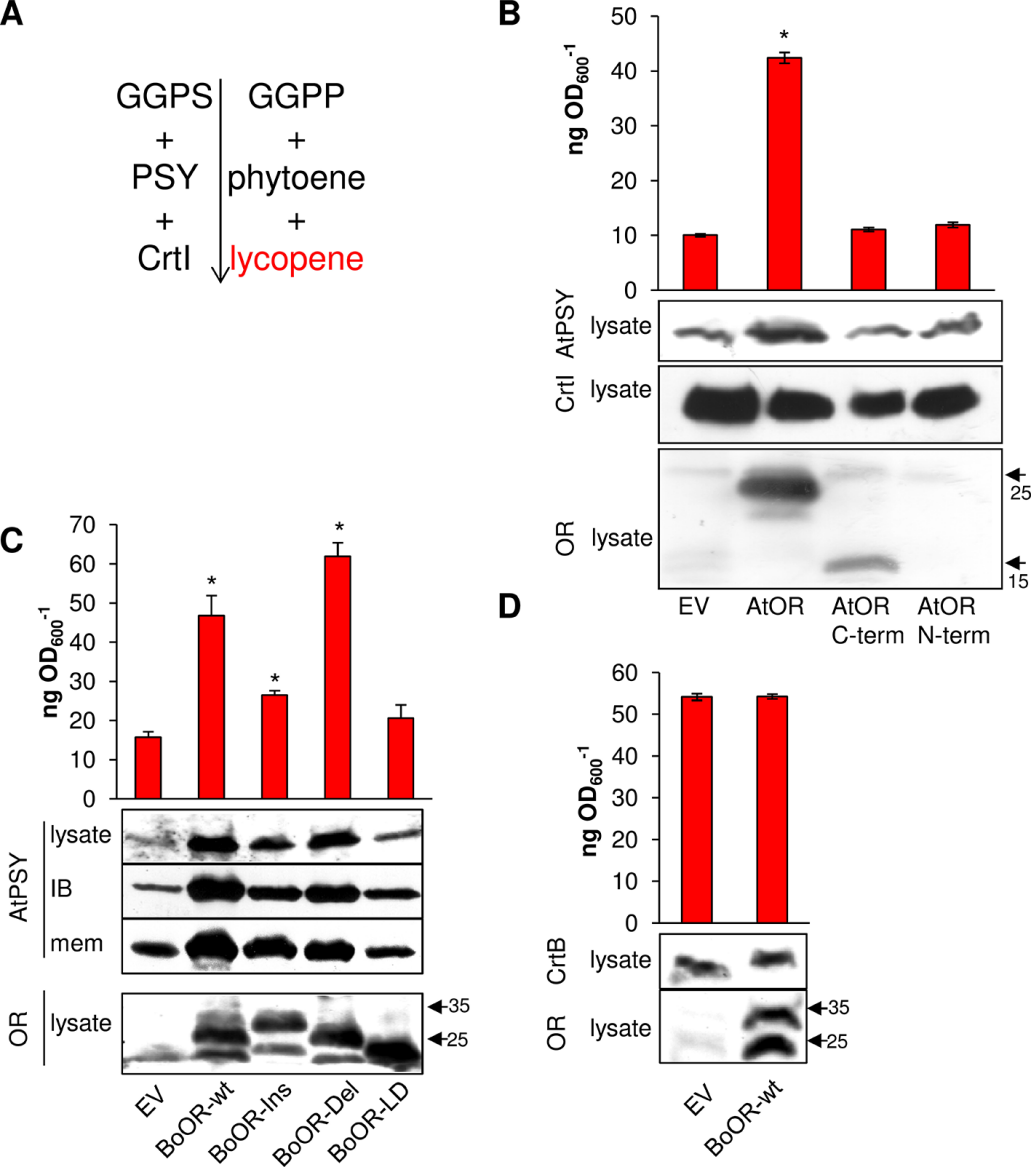
Heterologous expression of OR variants in *E. coli* expressing a mini-pathway for lycopene synthesis. **(A)** The mini-pathway contains Arabidopsis GGPP synthase 11 (GGPS), Arabidopsis PSY and the bacterial phytoene desaturase CrtI, which catalyze the formation of GGPP, phytoene, and then red-colored lycopene in *E. coli,* respectively. Carotenogenic enzymes are shown on the left while intermediates/products are on the right side of the mini-pathway. **(B)** Coexpression of AtOR as well as N- and C-terminal AtOR moieties in lycopene-producing *E. coli* cells. Western blot with anti-OR antibody confirmed the presence of full-length and the C-terminal AtOR moiety (the epitope is located in the C-terminal moiety). Western blot with anti-CrtI antibodies confirmed specificity of the response for PSY. **(C)** Coexpression of BoOR-wt and BoOR mutant variants. Increased AtPSY levels affected both the population of AtPSY in insoluble and enzymatically inactive inclusion body and in the membrane fraction correlating with enzyme activity/lycopene levels. Western blot with anti-OR antibodies shows all BoOR variants expressed at similar levels in *E. coli*. IB, inclusion body fraction; mem, membrane fraction. **(D)** Coexpression of BoOR-wt in a mini-pathway in which AtPSY was replaced by the bacterial phytoene synthase CrtB. A western blot with anti-CrtB antibodies is shown below. The empty vector (EV) was included as negative control. Protein marker bands are denoted with arrows. Lycopene was quantified during exponential growth of *E. coli* and is expressed in ng lycopene per OD_600_. Results are means ± SD from three biological replicates. *Significant difference (P < 0.05).

Expression of BoOR mutant variants in yeast and Arabidopsis revealed differences regarding heterodimerization capacity (**Figure 2**), interaction capacity with AtPSY (**Figure 3**), and impact on their protein expression in Arabidopsis (**Figure 4**). *E. coli* provides an additional system that is capable of producing carotenoids when proper carotenogenic enzymes are co-expressed. For instance, *E. coli* expressing the carotenogenic mini-pathway (**Figure 6A**) containing both Arabidopsis GGPP synthase 11 and Arabidopsis PSY as well as the bacterial phytoene desaturase CrtI from *Pantoea ananatis* generate red-colored lycopene. The lycopene produced in *E. coli* can be quantified photometrically, which provides a convenient read-out corresponding to PSY enzymatic activity as phytoene is readily and fully converted by CrtI (Camagna et al., 2019). This system was successfully used to determine PSY activity differences caused by single nucleotide or amino acid polymorphisms (Welsch et al., 2010; Mlalazi et al., 2012; Cao et al., 2019).

To investigate whether this system was also suitable to examine the effect of OR on PSY, we first co-expressed AtOR in *E. coli* harboring the carotenogenic mini-pathway AtGGPS11, AtPSY, and CrtI for lycopene production. Remarkably, *AtOR* co-expressing *E. coli* showed increased AtPSY protein levels revealed by western blots and increased AtPSY activity, i.e. lycopene formation (**Figure 6B**). This is in accordance with observations in Arabidopsis *AtOR*-overexpressing calli and roots, in which PSY protein levels were increased and resulted in increased total carotenoid content (Zhou et al., 2015). This suggests that the interaction between AtOR and AtPSY took place also in this heterologous system and the increase of AtPSY protein stability resulted in increased activity and lycopene accumulation. Co-expression of only the N- or C-terminal moiety of AtOR did not result in any changes in AtPSY protein and lycopene level in *E. coli* (**Figure 6B**). This is because OR dimerization requires the C-terminal DnaJ-like zinc finger domain while interaction with PSY requires the N-terminal moiety. This result corroborates that the combined action of both moieties is required to posttranslationally stabilize PSY.

We next co-expressed *BoOR* mutant variants in the lycopene-producing *E. coli* and compared both AtPSY protein levels and lycopene production (**Figure 6C**). While BoOR-wt led to increased lycopene levels like the strain coexpressing *AtOR* (compare **Figure 6B**), lycopene in the *BoOR-Del* coexpressing strain was even higher while coexpression of *BoOR-Ins* showed a lower but significant increase compared with the control. In contrast, lycopene in *BoOR-LD* coexpressing *E. coli* was similar to the empty vector control. When we checked bacterial subfractions for the presence of AtPSY by western blot analysis, we detected AtPSY protein in the membrane and inclusion body fractions, while the cytoplasmic fraction was devoid of any AtPSY signals. The inclusion body fraction corresponds to a population of insoluble, aggregated AtPSY that is enzymatically inactive (Camagna et al., 2019). Interestingly, while coexpression of *BoOR-wt, BoOR-Ins* and *BoOR-Del* increased AtPSY levels in both fractions, the relative increase in comparison to the empty vector control was more pronounced in the inclusion body fraction (**Figure 6C**). Moreover, the relative increases in AtPSY protein levels observed in the membrane fractions corresponded to the relative increases in lycopene production in *E. coli* coexpressing the corresponding *BoOR* variants. The variations of PSY protein levels and lycopene accumulation among the variants were not correlated with BoOR protein levels (**Figure 6C**).

To investigate whether the posttranslational effect of OR on PSY was restricted to plant-type phytoene synthases, Arabidopsis PSY in the mini-pathway was replaced by the bacterial phytoene synthase CrtB. CrtB shares 61% similarity with Arabidopsis PSY and its coexpression with AtGGPS11 and bacterial CrtI led to lycopene formation (**Figure 6D**). Interestingly, coexpression of *BoOR-wt* neither affected CrtB protein amounts nor lycopene levels in corresponding bacteria compared with the empty vector control. This result indicates that BoOR specifically interacts with plant-type PSY to posttranslationally affect its stability and activity.

## Discussion

Overexpression of cauliflower *BoOR-mut* gene increased carotenoid levels in non-green tissues (Lopez et al., 2008; Li et al., 2012). However, the detailed molecular mechanisms underlying the action of *BoOR* mutant variants with different in-frame insertions and deletions are unknown. We investigated individual *BoOR* variants in detail by examining their heterodimerization activity with wild-type OR proteins, interaction capacity with PSY, protein stability *in planta,* and effects of individual expression on carotenoid accumulation in Arabidopsis as well as in a heterologous system. Our results suggest different properties provoked by the OR variants, which are associated with their effects on OR dimerization and PSY stability.

### Properties of BoOR Variants

Different functional properties can be attributed to the OR protein: while the N-terminal moiety interacts with PSY, the C-terminal moiety is responsible for OR dimerization (Zhou et al., 2015; Chayut et al., 2017). Furthermore, transmembrane predictions suggested the presence of two centrally located transmembrane domains (**Figure 1**). The existence of two transmembrane domains led to a membrane topology of the protein with both the PSY-interacting N-terminus as well as the C-terminal dimerization domain positioned on the same side of plastid membranes, while only a short loop connecting the two transmembrane domains is on the opposed membrane side (**Figure 1**). Although OR is reported to be found in the nucleus of etiolated Arabidopsis seedlings (Sun et al., 2016; Sun et al., 2019), OR is predicted to be a plastid-localized protein and was shown to be imported into plastids by GFP fluorescence co-localization in adult plants (Lu et al., 2006). Given its interaction with PSY that is reported to be localized in the thylakoid membrane in its active form (Bonk et al., 1997; Welsch et al., 2000; Shumskaya et al., 2012; Lätari et al., 2015), we assume OR is present in the thylakoid/stroma and/or envelope/stroma side with the N-terminus facing the stroma side.

Both the short insertion and deletion as in BoOR-Ins and BoOR-del, respectively, did not drastically alter the positioning of the functional interaction domains. Accordingly, expression of these *BoOR* mutant variants in both plants and *E. coli* did not show a significantly altered effect on correlated increase of carotenoid accumulation compared with the control expressing *BoOR-wt* due to similar increased PSY protein level and activity (**Figures 4** and **6**). These results are confirmed molecularly by unchanged heterodimerization and PSY interaction as examined in the split-ubiquitin system (**Figures 2** and **3**).

In contrast, the long deletion present in BoOR-LD eliminates the N-terminal transmembrane domain, which alters the membrane topology of BoOR such that the PSY-interacting N-terminus and the C-terminal dimerization domain are localized on opposite sides of the membrane (**Figure 1**). Accordingly, BoOR-LD homodimerization as well as heterodimerization with BoOR-wt is impaired. Absence of dimerization in BoOR-LD did not affect protein stability of BoOR-LD in the bacterial system, which is concluded from similar BoOR protein amounts as other BoOR variants upon coexpression (**Figure 6C**). However, absence of dimerization has dramatic consequences on the protein stability in a plastid environment in plants. While all BoOR variants with proper positioning of the zinc finger domain allowed BoOR proteins to accumulate in response to their gene expression, absence of dimerization in BoOR-LD resulted in greatly reduced protein levels despite high transcript levels (**Figures 4B–D**). This indicates that dimerization of OR is a major property specifically determining OR stability and amounts in plants. Protein homo- and heterodimerizations are assumed to stabilize proteins and thus prevent their degradation, as shown for 1-aminocyclopropane-1-carboxylate synthase and 14-3-3 proteins (Mei et al., 2005; Messaritou et al., 2010; Lee et al., 2017). As BoOR-LD instability is selectively observed in plastids but absent in *E. coli*, we suggest involvement of plastid-specific proteolytic components in its degradation.

### OR Functions in Heterologous Bacterial System

Coexpression of BoOR-wt as well as BoOR-Ins and BoOR-Del with proper localization of the PSY-interacting and dimerization domains had a similar effect on PSY protein stability in *E. coli* as in plants. This finding indicates that the holdase function of OR (Park et al., 2016) acts in *E. coli* too. Recombinant AtPSY was distributed in inclusion body fraction and in bacterial membranes, while the cytoplasm is devoid of detectable amounts of AtPSY. The membrane-localized fraction represents the enzymatically active fraction, whereas the inclusion body fraction corresponds to aggregated and thus enzymatically inactive PSY (Camagna et al., 2019). *OR* co-expression in *E. coli* increased the total AtPSY protein amounts as well as the carotenoid biosynthesis activity, as determined by higher lycopene levels (**Figure 6**). This correlates with increases in AtPSY protein in both the membrane and the inclusion body fraction although the large relative increases in the inclusion body fraction do not contribute to the increased pathway activity.

The current model of protein fates after their release from ribosomes assumes that nascent polypeptides face four major and competing pathways: i) they might be immediately degraded through proteolysis, ii) they might form aggregates, iii) they might spontaneously fold properly, and iv) they might bind to chaperones which release the native, properly folded protein (Fink et al., 1999). Coexpression of a PSY-specific chaperone – AtOR, BoOR-wt and its functional mutant variants - apparently strongly shifts the amount of properly folded AtPSY and thus initially its relative amounts in the bacterial membrane compared with the control with only host-derived chaperones. Because OR functions as a holdase, it is likely that OR interacts with properly folded AtPSY (either spontaneously or generated through host-derived chaperones) and extends its turnover. Subsequent unfolding might secondarily increase the amounts of aggregated AtPSY in the inclusion body fraction. This action of OR is similar to the widespread strategy of increasing the yield of properly folded, recombinant proteins in prokaryotic systems by coexpression of major cytoplasmic chaperones, namely, DnaK and GroEL, alone or in combination with their co-chaperones (Martínez-Alonso et al., 2010). However, the difference is in the specificity of OR for plant PSY folding maintenance, as we did not observe altered protein levels for bacterial CrtB (**Figure 6B**).

Although interaction with PSY is still present if the C-terminal dimerization domain is misplaced as in BoOR-LD, lack of OR heterodimerization in this variant prevents its proper function in maintaining PSY folding. Accordingly, PSY protein is neither increased in bacterial membranes nor in inclusion body fraction when BoOR-LD is expressed and consequently there is no increase in lycopene formation (**Figure 6C**). This observation finally characterizes BoOR-LD as a non-functional OR version and confirms that both OR dimerization and PSY interaction are required for full OR and PSY function.

### Putative Roles of BoOR Variants in Carotenoid Accumulation

Our findings show that two of the three BoOR mutant variants – BoOR-Del and BoOR-Ins – remain functional regarding PSY interaction and OR dimerization as well as increased carotenoid accumulation in Arabidopsis, whereas BoOR-LD is non-functional. This explains why expression of *BoOR-LD* showed unchanged carotenoid levels in seed-derived Arabidopsis calli.

However, interestingly compared to carotenoid increases observed with individual BoOR mutant variant expression, expression of the *BoOR-mut* allele that simultaneously produces various BoOR mutant variants resulted in higher carotenoid levels in Arabidopsis calli. Similarly, expression of the *BoOR-mut* allele also induces carotenoid overproduction in potato (Lopez et al., 2008). As concluded from our studies, BoOR-LD did not directly contribute to these observations, as its function was impaired due to domain mislocation. Presumably, the more pronounced effect observed when the *BoOR-mut* allele is expressed compared with individually expressed mutant variants on carotenoid accumulation suggests an additive effect that might occur through heterodimers of various BoOR mutant variants.

## Data Availability Statement

The datasets generated for this study are available on request to the corresponding authors.

## Author Contributions

RW, XZ, and LL designed the research. RW and XZ performed most of the experiments. JK, TSc, HY, and TSu carried out and/or contributed to some analyses. RW and LL wrote the article.

## Funding

This research was supported by funds from the HarvestPlus (http://www.harvestplus.org/) research consortium (grant 2014H6320.FRE) to RW and of the Deutsche Forschungsgemeinschaft (grant WE4731/3) to JK, as well as by funds from an Agriculture and Food Research Initiative competitive award grant no. 2016-67013-24612 from the USDA National Institute of Food and Agriculture to LL. The article processing charge was funded by the German Research Foundation (DFG) and the University of Freiburg in the funding programme Open Access Publishing.

## Conflict of Interest

The authors declare that the research was conducted in the absence of any commercial or financial relationships that could be construed as a potential conflict of interest.
