# Supplementary material for: Characterization of Cauliflower OR Mutant Variants
Source: Front Plant Sci. 2020 Jan 21;10:1716. doi: 10.3389/fpls.2019.01716 (PMC6985574; doi:10.3389/fpls.2019.01716)
Supplement: Supplementary file 1 [file DataSheet_1.pdf]

Supplemental Figure S1

BoOR wild type

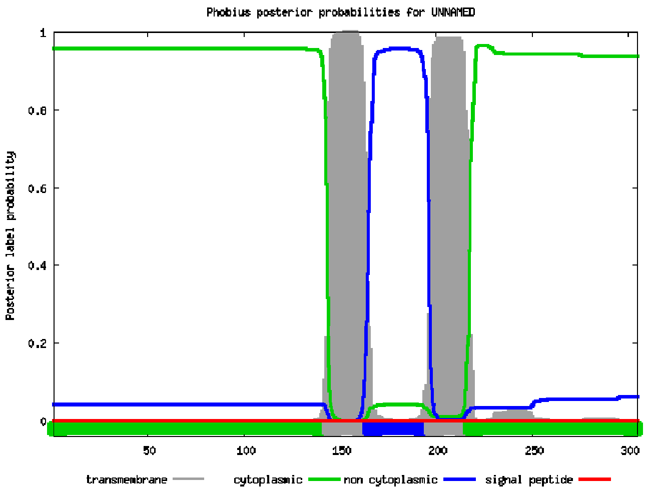

FT	TOPO_DOM	1	143	PLASMATIC.
FT	TRANSMEM	144	164	
FT	TOPO_DOM	165	196	NON-PLASMATIC.
FT	TRANSMEM	197	217	
FT	TOPO_DOM	218	305	PLASMATIC.

BoOR-Ins

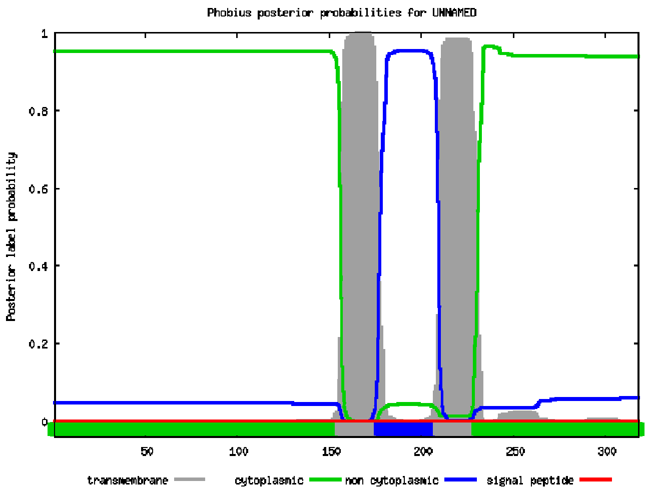

FT	TOPO_DOM	1	156	PLASMATIC.
FT	TRANSMEM	157	177	
FT	TOPO_DOM	178	209	NON-PLASMATIC.
FT	TRANSMEM	210	230	
FT	TOPO_DOM	231	318	PLASMATIC.

BoOR-del

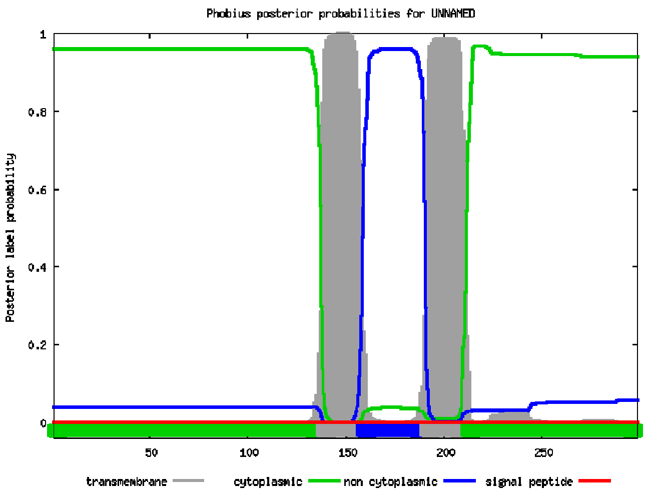

FT	TOPO_DOM	1	137	PLASMATIC.
FT	TRANSMEM	138	158	
FT	TOPO_DOM	159	190	NON-PLASMATIC.
FT	TRANSMEM	191	211	
FT	TOPO_DOM	212	299	PLASMATIC.

BoOR-LD

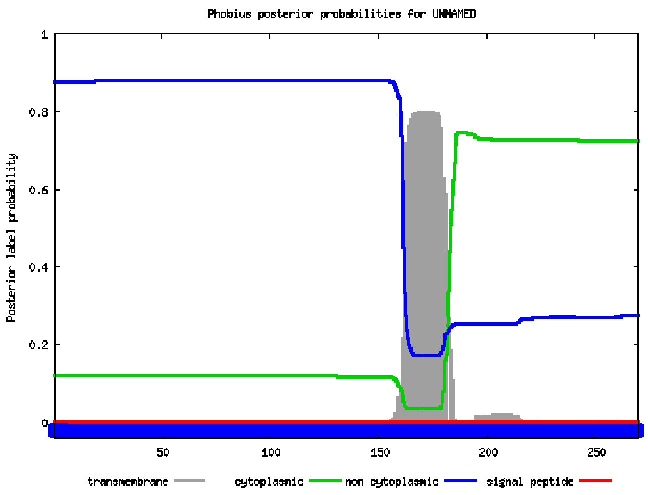

FT	TOPO_DOM	1	270	NON-PLASMATIC.

Supplemental Figure S1: Transmembrane predictions for BoOR variants
Transmembrane domains were predicted for the BoOR variants using the Phobius tool.

Supplemental Figure S2

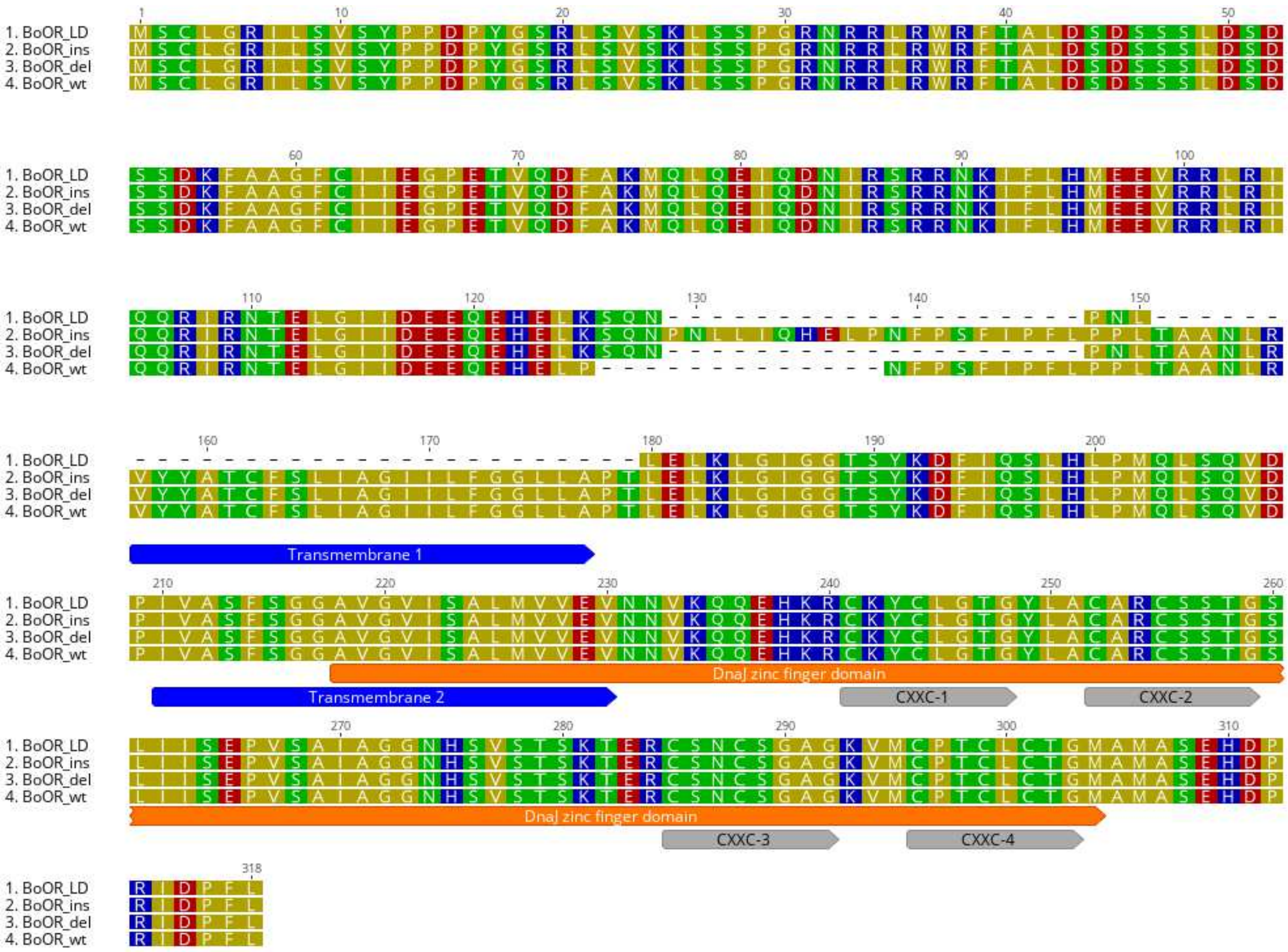

Supplemental Figure S2: Amino acid alignment of BoOR variants
Protein sequences of BoOR variants were aligned using the Blosum62 alignement score matrix with Geneious. The DnaJ zinc finger domain (orange arrow) contains eight conserved cysteine residues that bind to two zinc ions. The four CXXC motifs (CXXC-1 to CXXC-4, grey arrows) and the two transmembrane domains (blue arrow) are indicated. Acid amino acids (red), basic amino acids (blue), neutral amino acids (yellow), uncharged amino acids (green).

Supplemental Table S1

Use	Target Gene/vector/accession	Primer Sequence	TP
Y2H	BoOR-del/pRTL2-BoOR-del/DQ_482458	B1-TCCGACAAATTCGCTGCCGGCTTTTG/ B2-AAGGAAGGGATCAATACGAGGGTCG	54
Y2H	BoOR-ins/pRTL2-BoOR-ins/DQ_482457	B1-TCCGACAAATTCGCTGCCGGCTTTTG/ B2-AAGGAAGGGATCAATACGAGGGTCG	54
Y2H	BoOR-ins/pRTL2-BoOR-LD/DQ_482459	B1-TCCGACAAATTCGCTGCCGGCTTTTG/ B2-AAGGAAGGGATCAATACGAGGGTCG	54
GFP	BoOR-del/pRTL2-BoOR-del/DQ_482458	aaaagaattcATGTCTTGTTTGGGTAGGATC /aaaacccatggaAAGGAAGGGATCAATACG AGG	
GFP	BoOR-ins/pRTL2-BoOR-ins/DQ_482457	aaaagaattcATGTCTTGTTTGGGTAGGATC /aaaacccatggaAAGGAAGGGATCAATACG AGG	
GFP	BoOR-ins/pRTL2-BoOR-LD/DQ_482459	aaaagaattcATGTCTTGTTTGGGTAGGATC /aaaacccatggaAAGGAAGGGATCAATACG AGG	

With B1: ACAAGTTTGTACAAAAAAGCAGGCTCTCCAACCACCATG

B2: TCCGCCACCACCAACCACTTTGTACAAGAAAGCTGGGTA

Supplemental Table S1: Primers used in this work
TP indicates the transit peptide (in amino acids) which is eliminated upon expression in the corresponding vectors; Y2H, constructs for expression in yeast two-hybrid system; GFP, constructs for expression of GFP fusion proteins in Arabidopsis.
